# Facial Contouring Through Jaw Exercises: A Report of Two Cases Examining Efficacy and Consumer Expectations

**DOI:** 10.7759/cureus.74635

**Published:** 2024-11-27

**Authors:** Kritin K Verma, Ryan Koch, Karan Gidwani, Brooke Walterscheid, Daniel P Friedmann, Palak Parekh, Michelle Tarbox

**Affiliations:** 1 School of Medicine, Texas Tech University Health Sciences Center, Lubbock, USA; 2 School of Medicine, Texas A&M University, College Station, USA; 3 Osteopathic Medicine, Touro University Nevada, Henderson, USA; 4 Dermatology, Texas Tech University Health Sciences Center, Lubbock, USA; 5 Dermatology, Westlake Dermatology Clinical Research Center, Westlake Dermatology and Cosmetic Surgery, Austin, USA; 6 Dermatology, Baylor Scott and White Medical Center, Temple, USA

**Keywords:** consumer products, deceptive advertising, efficacy, facial contouring, jaw exercises

## Abstract

This study examines the efficacy of jaw exercising products for facial contouring. The two individuals used a commercially available jaw exerciser for approximately three months, following the provided instructions. Neither case reported noticeable changes in jaw appearance based on subjective measurements. In this study, a review of scientific literature found limited evidence supporting these devices' ability to reduce double chins, enhance jawlines, or tighten facial skin. The mastication muscles targeted by these exercises do not directly affect submental fat or skin elasticity. While some studies show potential benefits for facial rejuvenation with specific devices, the overall evidence for jaw exercisers remains inconclusive. The results from the cases highlight concerns about potentially misleading marketing claims and emphasize the need for larger, more objective studies to definitively assess these products' effectiveness. Limitations include the small sample size and the potential for bias. The findings suggest that jaw exercising products may have limited efficacy in achieving advertised results for facial contouring.

## Introduction

The growing popularity of jaw exercising products, which are frequently advertised on venues, such as Amazon and social media, has sparked tremendous consumer interest in their potential advantages for facial contouring. These products promise to improve face appearance through jaw exercises.

Despite these advertising claims, there is limited scientific data to support the ability of these devices to reduce double chins, enhance jawline structure, or result in face and neck tightening. Previous studies on facial workouts produced conflicting outcomes. For example, a control group study evaluating the effectiveness of facial workouts for reducing wrinkles and sagging discovered no significant differences between the experimental and control groups, implying that facial exercises may not be useful for face rejuvenation [1]. Furthermore, a systematic assessment of exercise therapy for temporomandibular disorders (TMDs) found that while some exercises can increase jaw mobility and reduce discomfort, their effect on face contouring is unknown [2].

We analyzed the efficacy of jaw exercise tools through a report of two cases. Additionally, a literature review was done on the masticatory muscles involved, including the temporalis, masseter, medial pterygoid, and lateral pterygoid [3]. The findings will add to the continuing debate over the efficacy of these products and their potential for deceptive advertising.

## Case presentation

The two cases in this report individually experimented with a set of jaw-exercising products at independent times at their leisure, due to interest in enhancing their jawline as advertised online. The jaw-exercising products, which were obtained online for around $10 each by the individuals in this report, were used according to the instructions provided with the product at the time of purchase for approximately 10 minutes, one to three times per week. The participants used the product for roughly three months. These patients individually documented the course of their jaw-exercising treatment by taking images before and after.

**Figure 1:**
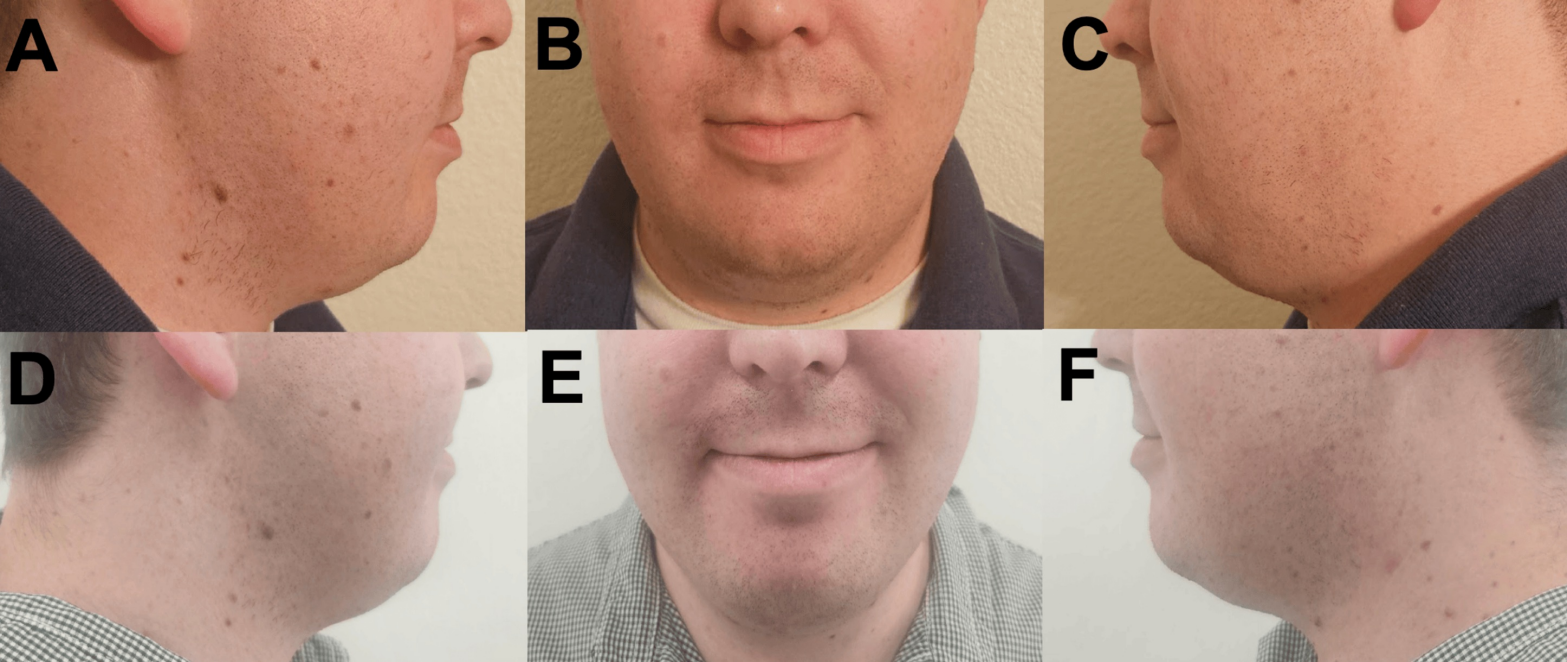
Before (A-C) and after (D-F) pictures of the patient in Case 1.

Case 1 involved a 27-year-old male with a BMI of 30.0 who used the product two to three times per week for three months. The patient's diet and exercise were noted to be the same during this period as before the initiation of the jaw products. Exact details on exercise frequency and diet were not obtained. Before and after pictures were taken of the patient’s face (Figures 1A-1F). The participant reported no statistically significant change in jaw appearance and facial contouring.

**Figure 2:**
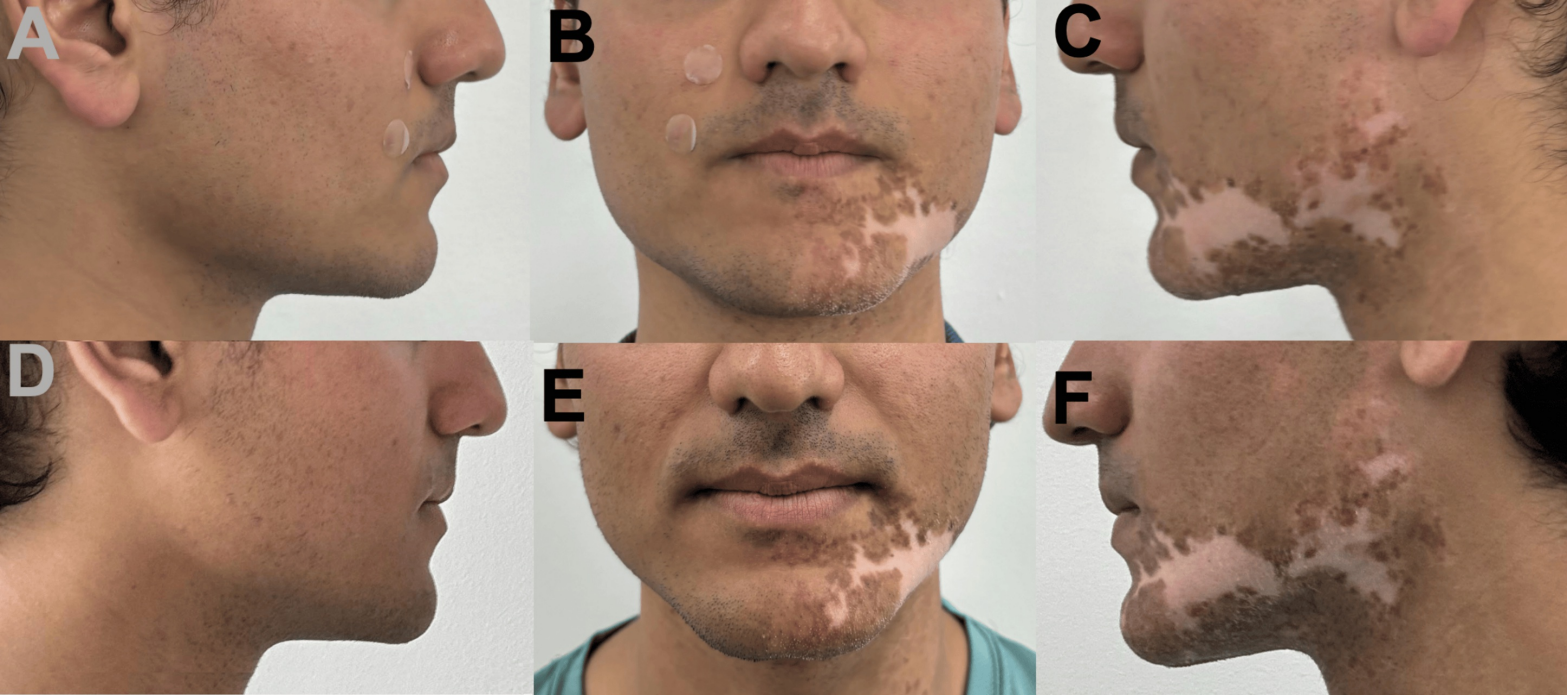
Before (A-C) and after (D-F) pictures of the patient in Case 2.

Case 2 was a 24-year-old male with a BMI of 24 who used the product one to two times per week for three months. The patient reported being active three to four times a week before and during the use of the jaw exerciser. The patient noted no vast change in diet intake during the use of the product and stated that it was the same as before the initiation of the exerciser. Before and after pictures were taken of the patient’s face (Figures 2A-2F). The second patient had vitiligo, which may make it harder to evaluate facial contouring; however, did not impact the findings from the analysis, which indicated no statistically significant change in observable facial contouring.

Various criteria were evaluated including facial contouring changes and overall satisfaction between patients (Table 1). The decision for documented facial contouring was a unanimous decision between the participants themselves and all the authors of this paper. The results for overall satisfaction were the self-documented experience with the product themselves and were reported as either very dissatisfied, slightly dissatisfied, neutral, slightly satisfied, and very satisfied. Neither case saw any change in the reduction of the double chin line, jawline enhancement, as well as face and neck tightening.

**Table 1:** Evaluation of device and results from participants.

Evaluated criteria		Case 1	Case 2
Documented facial contouring changes	Double chin reduction	No change	No change
	Jawline enhancement	No change	No change
	Face and neck tightening	No change	No change
Overall satisfaction	Ease of use	Neutral	Slightly satisfied
	Easy to clean	Neutral	Slightly dissatisfied
	Suctioning problem	Very dissatisfied	Slightly dissatisfied
	Gagging	Slightly dissatisfied	Very dissatisfied
	Unstable in mouth	Slightly dissatisfied	Neutral
	Rubbery feeling	Slightly dissatisfied	Slightly dissatisfied

## Discussion

Jaw exercisers typically target the mastication muscles, which control chewing movements [3]. These muscles - temporalis, masseter, medial pterygoid, and lateral pterygoid - have no direct effect on submental double chin fat reduction, jawline enhancement, or skin tightening based on the three cases we reported in this study [3]. This understanding is supported by scientific literature. For example, a study on face exercises discovered no significant increase in facial rejuvenation, implying that activities aimed at facial muscles may not successfully eliminate wrinkles or sagging skin [1]. Similarly, assessments of exercise therapy for TMDs found that while exercises can increase jaw mobility and reduce discomfort, they have no meaningful effect on face contours [2,4,5].

One study concluded that facial muscle exercises could lead to beneficial effects on facial rejuvenation [6]. However, the device used in this study had a vastly different mechanism than the one used in the present study [6]. The Pao device (Nagoya, Japan: MTG Co., Ltd), which is held in the mouth and moves in an oscillating motion by nodding the head, was used for 30 s twice a day for a total of eight weeks [6]. The researchers found that the exercises led to changes in facial surface distances, surface areas, and volumes resulting in decreased facial wrinkles and jawline sagging [6]. The Pao device targets facial muscles differently than the static resistance provided by jaw exercisers in our cases [6]. This distinction in the mechanism of action could explain the disparity in outcomes.

Weight gain, genetics, and aging are common causes of double chin reduction that cannot be treated with muscle exercises alone [1]. There is no strong scientific data to support the effectiveness of jaw workouts in reducing submental fat. While workouts can improve jaw muscle strength, they do not affect the underlying bone structure or facial shapes. Non-surgical procedures such as fillers and botulinum toxin (Botox) injections have shown greater potential in improving facial esthetics [1]. Skin tightening is influenced by skin elasticity and fat distribution. Exercises that target the jaw muscles may not have any meaningful effect on these parameters [1]. Furthermore, recent advancements in non-invasive facial contouring techniques, such as high-intensity focused ultrasound (HIFU) and radiofrequency therapy, offer alternative approaches that may be more effective than exercise-based methods for addressing issues like submental fat reduction and skin tightening and should be considered for any patients considering facial contouring [7,8].

The fundamental limitation of this study is the subjectivity of the presented results. Quantifiable methods to measure facial contouring (e.g., using 3D imaging technology or facial scans yielding facial fat percentage) would have more precisely analyzed the efficacy of the product. Another limitation was that the BMIs for before and after product use were not obtained. Blinding patients in future studies may help eliminate bias in identifying the efficacy of jaw exercisers. Additionally, participants, who are also the authors, may have biases that influence their findings. Furthermore, the small sample size and absence of objective measurements make the findings qualitative and potentially biased. The marketing promises, which may be typically seen in online products, for these jaw exercisers need to be more supported by scientific research [9]. The products do not treat the underlying reasons for double chins or skin laxity; hence, they do not produce the expected outcomes. This raises issues regarding deceptive advertising, and customers should use caution when considering such products.

## Conclusions

According to the findings of this report of two cases, jaw exercising items have limited efficacy in terms of double chin reduction, jawline enhancement, and face and neck tightening. The findings indicate that these products may not achieve the stated results, and their benefits are mostly subjective and minor. More studies with larger sample sizes and objective metrics are required to verify their efficacy definitively.
